# Accuracy of shear wave elastography in characterization of thyroid nodules in children and adolescents

**DOI:** 10.1186/s13244-021-01074-7

**Published:** 2021-09-09

**Authors:** Mohammed Hazem, Ossama M. Zakaria, Mohamed Yasser Ibrahim Daoud, Ibrahim Khalid Al Jabr, Abdulwahab A. AlYahya, Ahmed Gaber Hassanein, Abdulrahim A. Alabdulsalam, Mohammed Qasem AlAlwan, Nahla Mohamed Ali Hasan

**Affiliations:** 1 Department of Surgery, College of Medicine, King Faisal University, P.O. Box: 400, Al-Ahsa, 31982 Kingdom of Saudi Arabia; 2 Department of Radiology, Sohag Faculty of Medicine, Sohag University, Sohag, Egypt; 3 Department of Surgery, Division of Pediatric Surgery, Faculty of Medicine, Suez Canal University, Ismailia, Egypt; 4 Radiology Department, Polyclinic Center, King Faisal University, P.O. Box: 400, Al-Ahsa, 31982 Kingdom of Saudi Arabia; 5 Albaha University Medical Center, Albaha, Kingdom of Saudi Arabia; 6 Maxillofacial Surgery Unit, Surgery Department, Sohag Faculty of Medicine, Sohag University, Sohag, Egypt; 7 Department of Biomedical Sciences, College of Medicine, King Faisal University, Al Ahsa, Kingdom of Saudi Arabia; 8 Department of Radiology, King Fahd Hospital Hofuf, Al Ahsa, Kingdom of Saudi Arabia; 9 Sohag University Hospital, Sohag, Egypt

**Keywords:** Ultrasound, Shear wave elastography, Thyroid nodules, Children and adolescents

## Abstract

**Background:**

Thyroid nodules are an important health problem in children and adolescents. They possess a higher risk of malignancy in comparison to adults. This fact forms a great dilemma for clinicians. The aim of this study was to evaluate the reliability of shear wave elastography (SWE) as a non-invasive technique in the characterization of thyroid nodules in children and adolescents.

**Methods:**

This prospective study included 56 patients with thyroid nodules. All the patients underwent clinical assessment, laboratory investigations, ultrasound, and Doppler examination, followed by an SWE assessment. Statistical analysis was performed and the best cut-off value to differentiate benign from malignant nodules was determined using the ROC curve and AUC.

**Results:**

Seventy-two nodules were detected in the examined patients (ages ranged from 11 to 19 years, with mean age of 14.89 ± 2.3 years). Fifty-eight nodules (80.6%) were benign, and fourteen nodules (19.4%) were malignant (histopathologically proved). Highly suspicious criteria for prediction of malignancy by ultrasound and Doppler were hypoechoic echopattern, internal or internal and peripheral vascularity, microcalcifications, taller-than-wide dimensions, irregular outlines, and absence of halo (*p* < 0.05). The diagnostic performance for their summation was 70.69% sensitivity, 82.8% specificity, 80.45% accuracy, a 63.79% positive predictive value (PPV), and 87.9% negative predictive values (NPV). Regarding SWE, our results showed that 42.2 kPa was the best cut-off value, with AUC = 0.921 to differentiate malignant from benign nodules; the diagnostic performance was 85.71% sensitivity, 94.83% specificity, 93.06% accuracy, 76.9% PPV, and 93.2% NPV.

**Conclusion:**

Shear wave elastography is a non-invasive technique that can assist in the diagnosis of malignant thyroid nodules among children and adolescents.

## Key points

- Shear wave elastography (SWE) is an innovative technique providing new insights into thyroid nodules characterization.
- Malignant thyroid nodules have higher SWE indices (stiffer) than benign nodules.
- SWE has better diagnostic performance than ultrasound and Doppler imaging.

## Introduction

Thyroid nodules in children pose a significant problem and dilemma for endocrinologists, surgeons, and radiologists in regard to their pathological nature [1, 2]. In general, thyroid nodules in adults are more likely to be benign rather than malignant. However, among children and adolescents, the chances of a nodule being malignant is higher in comparison to adults [3]. Many patients with thyroid malignancy present with local metastasis in the cervical lymph nodes. This might be attributed to delay in diagnosis. However, distant metastasis is not as common [4].

Many diagnostic tools exist for evaluating thyroid nodule/s. The universally accepted protocol includes a thorough clinical examination, laboratory investigations, thyroid sonography, as well as fine-needle aspiration biopsy (FNAB) [2].

There is a debate on which is the best tool to investigate thyroid nodules. Sonography is still considered to have a higher range of diagnostic sensitivity and specificity compared to clinical examination [5]. However, ultrasound features of thyroid nodules in children are not strong predictors of malignant or benign etiology of thyroid nodules [6]. FNAB is currently considered one of the most accurate techniques to assess the pathological nature of thyroid nodules. However, it may be difficult to perform in children [7].

The last decade has witnessed the increasing use of sonoelastography (SE) as an additional tool in the assessment of thyroid nodules [8]. SE relies on the concept that malignant tumors are usually stiffer and firmer compared to their benign counterparts [9]. Earlier SE techniques depended on assessing tissue stiffness by applying strain/ pressure upon the examined tissue; however, this technique does entail some limitations. These include low reproducibility, lack of quantitative assessment of tissue stiffness, as well as being operator dependent [10].

Shear wave elastography (SWE) is a relatively newly developed SE technique that can quantitatively evaluate the examined tissue for its stiffness. It measures elasticity by tracing the shear wave propagation through tissues to provide quantitative measurements. It has been reported to be more reproducible and less operator dependent in comparison to SE [11, 12]. The use of SWE for quantitative assessment of the elasticity of adult thyroid nodules has been previously reported. These reports showed that quantitative elastographic evaluation of thyroid nodules was significantly different between malignant and benign nodules [13–15]. Normal thyroid tissue elasticity in children was previously assessed [16]. However, very few studies have reported the use of elastography to assess thyroid nodules in children [17, 18], and, to the best of our knowledge, none have yet studied the use of SWE specifically for differentiating benign and malignant thyroid nodules in the pediatric population.

The current study aims to evaluate the reliability of SWE in characterizing thyroid nodules among children and adolescents through correlation with FNAB and histopathological results. Additionally, the study aims to establish a cut-off value to distinguish between benign and malignant thyroid nodules.

## Methodology

### Study design

This prospective study took place over a period of five years from June 2015 to May 2020. Ethical approval was obtained from our intuitional review board (IRB), and informed written consent was obtained from patients’ parents or legal guardians. All techniques were performed in accordance with the Helsinki Declaration 1975, as revised in 2013. The study included 56 children and adolescents referred from relevant outpatient clinics who clinically presented with nodular thyroid enlargement. Exclusion criteria included patients with completely cystic nodules, nodules with coarse calcifications, and patients that refused to do FNAB or surgical excision in cases with indeterminate FNAB results (which is considered the golden standard for diagnosis).

### Assessment of patients

All the patients were interviewed for clinical assessment and laboratory investigations, followed by multiple radiological examinations that included chest X-ray, abdominal ultrasound, and different thyroid ultrasound techniques, including B-mode ultrasound, Harmonic ultrasound, Doppler ultrasound, and SWE examination. FNAB was performed for all cases, and the final diagnosis was based on cytopathologic results in addition to histopathologic results of the surgically resected specimen. Moreover, all proved to be benign nodules by cytopathological, or histopathological analysis; they were subjected to clinical and ultrasound follow-ups to exclude any possibilities of false negative results.

### Conventional and Doppler ultrasound examination

Ultrasonographic examination was performed by a single expert radiologist who has an experience of 16 years in thyroid ultrasound and Doppler examination, using a Philips EPIQ 7G ultrasound machine (Philips, USA) with a 7–12 MHz broadband linear array transducer. Patients were examined in supine position with elevated shoulders by small pads to facilitate full extension of the neck that leads to complete exposure of the lower margins of the thyroid glands. Scans of the gland were obtained in both longitudinal and transverse planes. All thyroid lesions were initially assessed by B- mode and Doppler ultrasound.

**Fig. 1:**
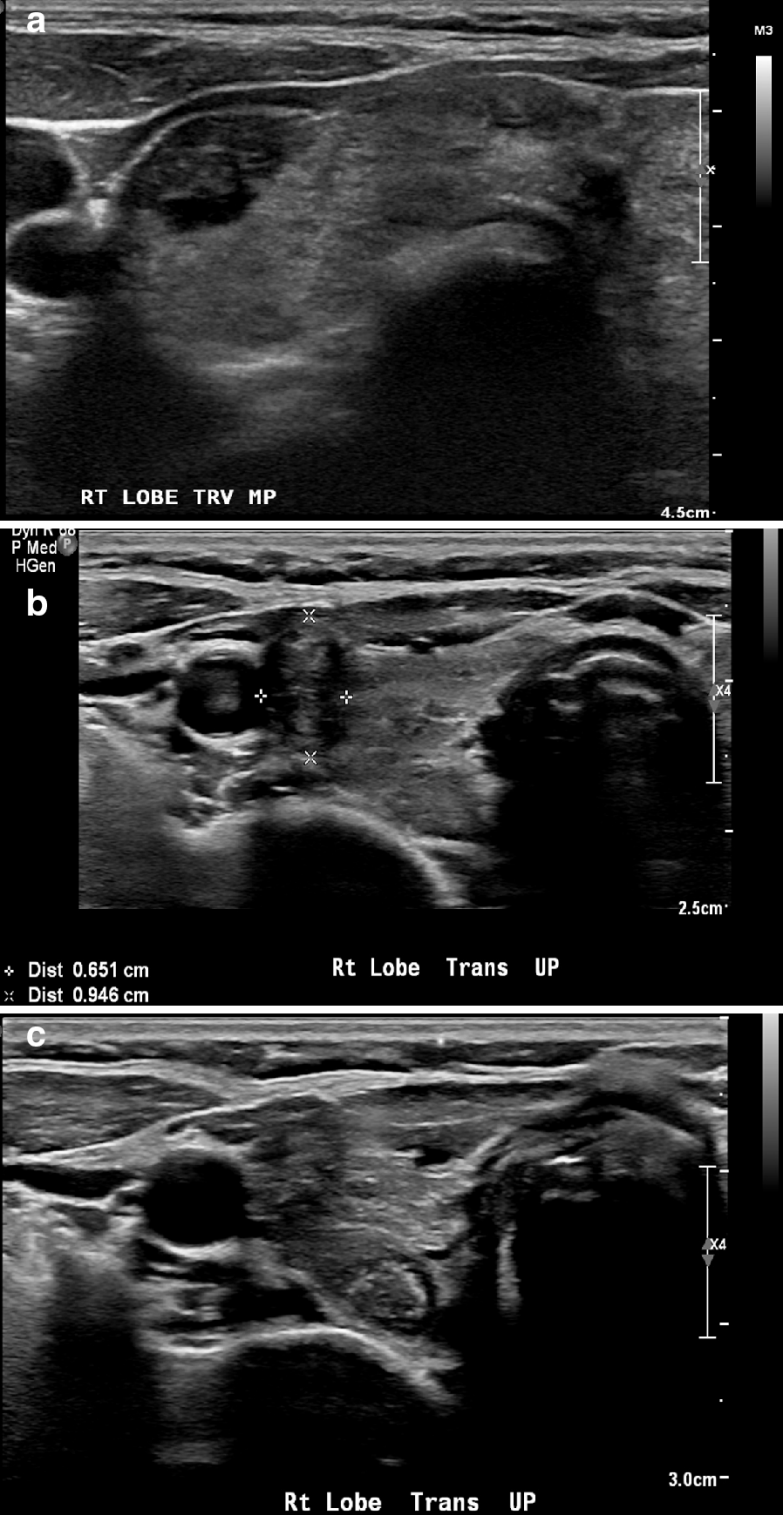
Multiple transverse B-mode ultrasound images obtained by linear probe examination showing: **a** Well-defined hypoechoic nodule with irregular outline within the right lobe of the thyroid gland. **b** Well-defined non-homogenous hypoechoic, taller-than-wide; smooth outline nodule is seen within the right thyroid lobe. It is not surrounded by a halo and does not contain internal calcification. **c** Well-defined isoechoic nodule surrounded by hypoechoic halo with regular outline within the posterior aspect of the right lobe of the thyroid gland

**Fig. 2:**
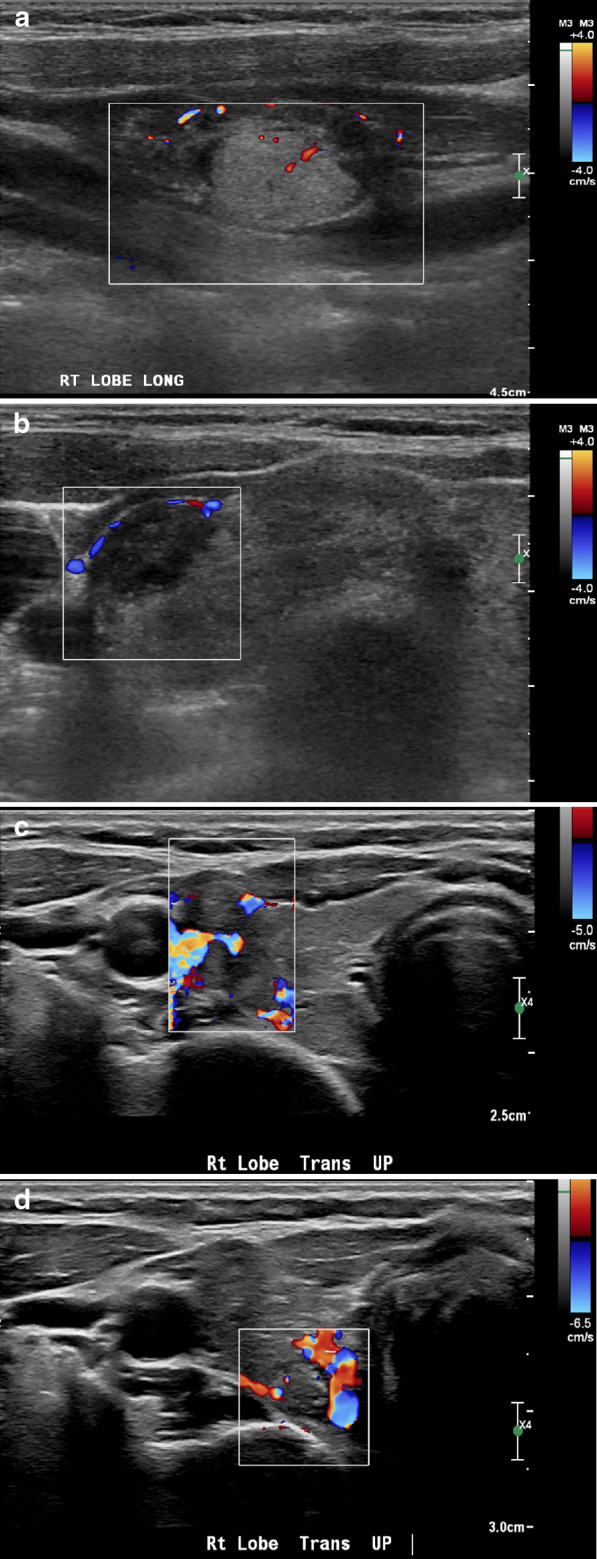
Multiple transverse and longitudinal Doppler ultrasound images performed by linear probe showing: **a** A hyperechoic nodule with internal vascularity seen within. **b** A hypoechoic nodule with peripheral vascularity seen surrounding it. **c** A non-homogenous isoechoic nodule with mixed peripheral and central vascularity. **d** A hyperechoic nodule with mixed peripheral and internal vascularity

The following parameters were assessed in each nodule, as recommended by the American Thyroid Association (ATA) guidelines: size; shape; margin; echogenicity (hypoechoic, isoechoic, and hyperechoic); internal content (completely solid or partially solid); presence or absence of calcifications, and, if present, its size was determined as microcalcification when < 1 mm or macrocalcification > 1 mm; and the presence of absence of a halo sign around the nodule (Fig. 1). Furthermore, the nodules were classified into two types according to their vascular patterns as assessed by Doppler ultrasound: Type 1: Absent blood flow within the nodule or the blood flow present only at the periphery of the nodule; Type 2: Blood flow detected within the center of nodule or within the center and around the nodule (Fig. 2).

Moreover, we implemented sonographic assessment of the internal cervical chain of the lymph nodes. This technique was significantly valuable in differentiating the hyperechoic and punctate calcifications due to papillary carcinoma from other malignant cervical lymphadenopathies [19].

### SWE examination

After completing ultrasonographic examination, SWE examination was performed during the same sitting by the same radiologist, who has five years of experience in SWE examination. The transducer was placed in a sagittal plane to avoid motion artifacts generated by the trachea and carotid arteries. Patients were asked to hold their breath and to not swallow for a short time while the image of the SWE was formed. The transducer was held in a stable position without performing any compression over the gland to minimize the artifacts resulting from compression (free hand technique), then SWE was started upon the conventional sonographic image.

The color box with adequate size was applied and displayed as an area of multiple colors, with blue representing softer tissue and red representing harder tissue. Images were obtained when there were no artifacts.

**Fig. 3:**
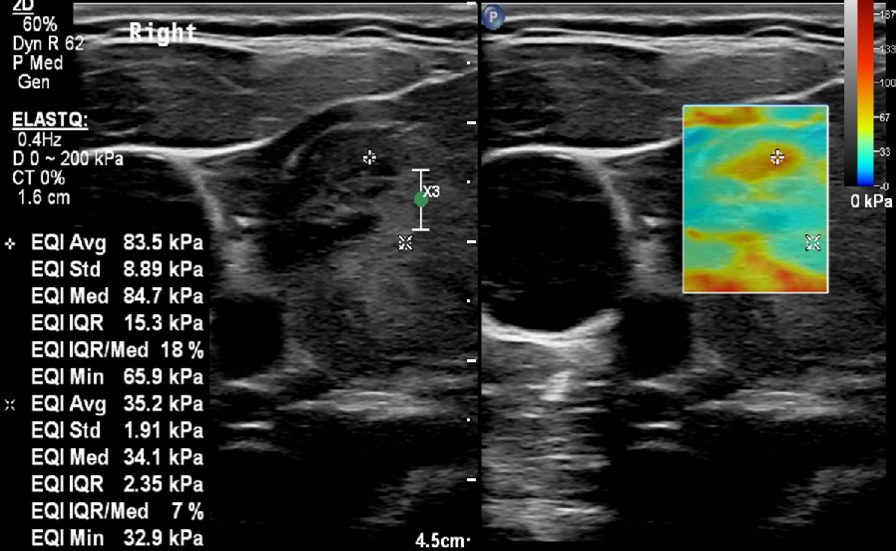
A 14.5-year-old female patient with FNAB-proven papillary thyroid carcinoma. Transverse scan for left lobe of the thyroid gland obtained by linear probe. On the right side of the figure, grayscale US shows a hypoechoic nodule with irregular margin which was assessed as a suspicious nodule on grayscale US. On the left side of the figure, SWE displays a heterogeneous color elasticity signal with central high SWE areas (red); mean SWE was 83.5 kPa

**Fig. 4:**
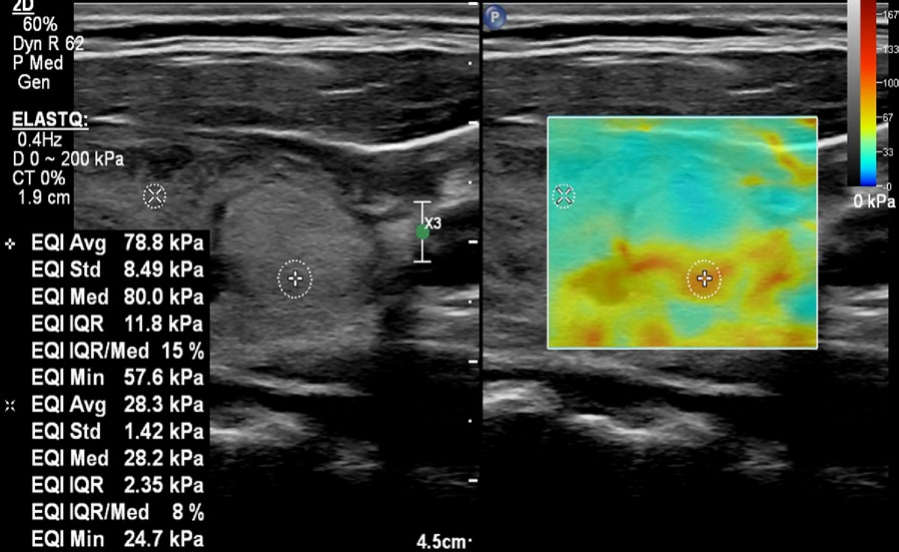
A 16-year-old male patient with FNAB-proven focal nodular hyperplasia. Transverse scan for right lobe of the thyroid gland performed by linear probe. On the right side of the figure, grayscale US shows a hyperechoic nodule with a well-defined margin. On the left side of the figure, SWE displays a heterogeneous color elasticity signal with peripheral high SWE areas (red)

Quantitative elastographic assessment was performed using a 2 X 2 mm region of interest (ROI), with the transducer positioned at the stiffest area, avoiding cystic areas, calcification, and normal thyroid tissue (Figs. 3, 4). All measurements were recoded and saved in kilopascal (kPa). Three measurements were taken for each nodule: the minimum, maximum, and mean SWE. The examination was repeated three times for each lesion, and the average of the three measurements was used as the final result.

### Fine-needle aspiration biopsy (FNAB) and histopathologic examination

Ultrasound-guided FNAB was taken from all patients for cytopathologic examination. The slides were stained with pap stain and a Romanowsky-type stain, and it was interpreted by a single expert pathologist. Reporting of FNAB results followed the Bethesda system for reporting thyroid cytopathology [20]. For those cases with indeterminate results (i.e., Category III and IV according to the Bethesda system) and who had surgical excision of the nodule performed, the final diagnosis was determined through histopathologic examination of the excised nodule.

### Statistical analysis

Data coding, entry, and analysis was accomplished with the aid of the SPSS® program (version 22) together with MedCalc® software. Descriptive statistics in the form of numbers and percentages were used for qualitative variables, while mean and standard deviation were used for quantitative variables.

For the detection of association and/or links between categorical grayscale ultrasonic characters and nature of thyroid nodules whether benign /malignant, a Chi-square test was used, and, when indicated, the Fisher exact test was used (with expected cell count less than 5). The odds ratio was calculated to assess these associations and the Phi co-efficient was calculated to determine the effect size. For these ultrasonic characters, the following were also calculated: the sensitivity, specificity, accuracy, PPV, and NPV.

The independent samples t-test was used to detect differences between malignant and benign nodules regarding quantitative SWE measures. For these quantitative SWE measures, a receiver operating characteristic curve was used to assess AUC, cut-off values, sensitivity, and specificity, in addition to calculating accuracy, PPV, and NPV. The accepted level of significance for all analyses was stated at 0.05 (two-tailed *p* value of < 0.05 was considered significant).

## Results

### Patients

Fifty-six patients were included in the study. They included 36 females and 20 males with a ratio of 1.8 to 1. Patients’ ages ranged from 11 to 19 years, with a mean age of 14.89 ± 2.3 years. Out of the 56 patients; 42 (75%) presented with a single nodule, while the remaining 14 (25%) had more than one nodule. The total number of nodules from all patients was 72.

### Pathological findings

Cytopathologic assessment along with histopathologic evaluation of excised nodules was considered as the main diagnostic method for the differentiation between benign and malignant nodules. On FNAB assessment, out of the 72 nodules, 51 (70.8%) were benign, 9 (12.5%) were malignant, and 12 (16.7%) showed indeterminate results. Cases with indeterminate FNAB results underwent surgical excision of the nodule, and the histopathologic assessment in these cases showed a benign result in seven cases and a malignant result in five cases. Therefore, fifty-eight (80.6%) nodules were benign, whereas 14 nodules (19.4%) proved to be malignant. All the malignant nodules were diagnosed as papillary carcinoma (n = 14). The majority of benign nodules were diagnosed as nodular hyperplasia/benign follicular nodules (n = 51, 87.9%). The remaining seven nodules (12.1%) were benign nodules associated with lymphocytic thyroiditis/Hashimoto thyroiditis (Table 1).

**Table 1:** FNAB and postoperative histopathology findings of thyroid nodules (n = 72 nodules in 56 patients)

FNAB and histopathology findings	Number	
Benign	Nodular hyperplasia/Benign follicular nodule	51
	Benign nodule associated with lymphocytic thyroiditis	7
	Total	58
Malignant	Papillary carcinoma	14
Total		72

### Conventional ultrasound and Doppler results

The mean size of thyroid nodules (according to the longest diameter) was (1.8 ± 0.68 cm; range: 0.8–3.2 cm) with no statistical significance between benign and malignant nodules. The other sonographic findings of the 72 nodules are variable and are mentioned in Table 2.

**Table 2:** Conventional ultrasound and Doppler criteria to detect malignant nodule ^(n=72)^

Ultrasound features	Malignant (n = 14)	Benign (n = 58)	Statistical significance		
	N (%)	N (%)	*p* value*	Odds ratio (95% CI)	Phi coefficiency #
*Echopattern*					
Hypoechoic	10 (71.4)	9 (15.5)	< 0.001	13.61 (3.49–53.03)	0.502
Not hypoechoic	4 (28.6)	49 (84.5)			
*Shape*					
Taller than wide	9 (64.3)	19 (32.8)	0.037	3.695 (1.09–12.55)	0.256
Wider than tall	5 (35.7)	39 (67.2)			
*Consistency*					
Solid	12 (85.7)	47 (81.0)	1.0	1.4 (0.27–7.2)	.048
Partially solid	2 (14.3)	11 (19.0)			
*Halo*					
Halo Absent	12 (85.7)	11 (19.0)	< 0.001	25.64 (5.0–131.44)	0.567
Halo Present	2 (14.3)	47 (81.0)			
*Outline*					
Irregular	8 (57.1)	8 (13.8)	0.002	8.333 (2.28–30.43)	.413
Regular	6 (42.9)	50 (86.2)			
*Calcification*					
Micro-calcification	11 (78.6)	2 (3.4)	< 0.001	102.67 (15.32–688.1)	0.773
No mico-calcification	3 (21.4)	56 (96.6)			
*Vascularity*					
Internal vascularity &/internal and peripheral	12 (85.7)	6 (10.3)	< 0.001	52.0 (9.32–290.11)	0.689
No vascularity and/peripheral vascularity	2 (14.3)	52 (89.7)			

*Fisher exact test was used as some expected cell values were less than 5

*CI*, Confidence interval

Significant *p* value if < 0.05 level

^#^ To detect effect size of the difference

Weak (0.2)–Moderate (0.4)–Strong (0.6)–Very strong (0.8)–Perfect (1.0)

The sonographic criteria which were commonly observed with malignant nodules included hypoechoic echopattern (observed in 71.4%), internal or internal and peripheral vascularity (85.7%), microcalcification (78.6%), and not surrounded by halo (85.7%). These criteria were statistically significant compared to their benign counterparts (*p* < 0.001 in all). Moreover, the malignant nodules were taller rather than being wide (64.3%) and had an irregular outline (57.1%); all these findings were statistically significant in comparison with benign nodules (*p* < 0.05) (Table 2).

The obtained statistically significant sonographic criteria were compared with pathological findings for the assessment of their diagnostic performance (sensitivity, specificity, accuracy, positive predictive value, and negative predictive values) as well as the assessment of the diagnostic performance for all these criteria combined (Table 3).

**Table 3:** Diagnostic performance of each conventional ultrasound and Doppler finding to detect malignant nodules ^(n = 72)^

Ultrasound features	Sensitivity % (95% CI)	Specificity % (95% CI)	Accuracy % (95% CI)	PPV % (95% CI)	NPV % (95% CI)
Hypoechoic echopattern	71.43 (41.9—91.61)	84.48 (72.58—92.65)	81.94 (71.11—90.02)	52.63 (35.88—68.81)	92.45 (84.16—96.58)
Taller than wide	64.29 (35.14—87.24)	67.24 (53.66—78.99)	66.67 (54.57—77.34)	32.14 (21.68—44.76)	88.64 (79.06—94.16)
Halo Absent	85.71 (57.19—98.22)	81.03 (68.59—90.13)	81.94 (71.11—90.02)	52.17 (38.08—65.93)	95.92 (86.62—98.84)
Irregular	57.14 (28.86—82.34)	86.21 (74.62—93.85)	80.56 (69.53—88.64)	50.0 (31.28—68.72)	89.29 (81.86—93.9)
Micro-calcification	78.57 (49.2—96.34)	96.55 (88.09—99.58)	93.06 (84.53—97.71)	84.62 (57.83—95.66)	94.92 (87.24—98.08)
Internal Vascularity	85.71 (57.19—98.22)	89.66 (78.83—96.11)	88.89 (79.28—95.08)	66.67 (47.65—81.46)	96. 30 (87.78—98.85)
Total*	94.2 (73.2–99.8)	69.89 (59.3- 81.85)	74.62 (61.7—85.9)	83.21 (54.5–95.9)	78.9 (63.7–87.6)

*PPV*, positive predictive value; *NPV* (negative predictive value

*Total include all above criteria

### Shear wave elastography (SWE) findings

Each nodule underwent minimum, maximum, and mean SWE measurements three times, and the average of these measurement was calculated for each index. All of the three measurements were higher among the malignant nodules compared to their benign counterparts with a *p* value of < 0.001 (Table 4).

**Table 4:** Mean values of SWE waves according to histopathology findings among the studied group

Parameter	Malignant	Benign	*p* value
SWE- Mean	49.71 ± 11.16	33.46 ± 5.02	< 0.001
SWE- Minimum	35.5 ± 8.79	22.19 ± 4.39	< 0.001
SWE- Maximum	63.91 ± 13.87	44.73 ± 5.89	< 0.001

*Independent samples t-test was used

The optimal cut-off value of the minimum, maximum, and mean SWE measurements to differentiate between benign and malignant nodules with the highest sensitivity, specificity, and accuracy was determined using receiver operating curve (ROC) analysis.

We identified the values of 42.2 kPa for mean SWE (area under curve, 0.921), 53.6 kPa for maximum SWE (area under curve, 0.895), and 26.9 kPa for minimum SWE (area under curve, 0.906) (Table 5) as the best cut-off values to distinguish between benign and malignant nodules. The diagnostic performance of each measurement is clarified in Table 6. Out of the three cut-off values, the mean SWE (42.2 kPa) shows the best sensitivity (85.71%), specificity (94.83%), accuracy (93.06%), and PPV (76.9%), while the maximum SWE (53.6 kPa) shows the best NPV (94.7%).

**Table 5:** Optimal cut-off values of SWE parameters

Parameter	AUC (95% CI)	Optimal cut-off values
SWE-mean	0.921 (0.832–0.971)	42.2
SWE-minimum	0.906 (0.814–0.962)	26.9
SWE-maximum	0.895 (0.801—0.955)	53.6

**Table 6:** Diagnostic performance of shear wave elastography to detect malignant nodules ^(n = 72)^

SWE	Sensitivity (95% CI)	Specificity (95% CI)	Accuracy (95% CI)	PPV (95% CI)	NPV (95% CI)
SWE- Mean	85.71% (57.2–98.2)	94.83% (85.6–98.9)	93.06% (85.5–96.8)	76.9% (51.3–91.3)	93.2% (85.7–96.9)
SWE- Minimum	71.43% (41.9–91.6)	86.21% (74.5–93.9)	83.34% (79.9–86.4)	55.6% (37.7–72.0)	92.6% (84.4–96.6)
SWE- Maximum	78..57% (49.2–95.3)	93.10% (83.3–98.1)	90.28% (83.2–94.4)	73.3% (50.7–88.0)	94.7% (86.8–98.0)

PPV (positive predictive value). NPV (negative predictive value)

Furthermore, we studied the diagnostic performance of the combined use of all ultrasound criteria mentioned in Table 3 and the mean SWE, and we found a significant increase in the diagnostic performance in comparison to using the ultrasound criteria only, but the diagnostic performance was variable when compared with mean SWE (sensitivity 96.3%, specificity 89.8%, accuracy 91.06%, PPV 90.06%, and NPV 88.3%).

## Discussion

Thyroid nodules, although uncommon among the pediatric population, pose a great challenge to pediatricians and pediatric surgeons. Among the pediatric population, the risk of a thyroid nodule being malignant may reach up to 18–26.7% [3].

Males and females are reported to have an equal proportion of thyroid malignancy in the pediatric age group. However, females may have a four-time prevalence risk compared to males at the post pubertal age [21]. The current study showed an apparently higher prevalence of thyroid nodule among females (64.3%) compared to their male peers (35.7%); this is concordant with previous studies [4, 22].

The current widely adopted methods of ultrasonographic differentiation between benign and malignant thyroid nodules rely mainly on certain sonographic criteria that have the highest summation of sensitivity and specificity as well as the highest accuracy [23, 24]. Many authors have reported that using a combination of multiple ultrasound criteria are necessary for diagnosis, and they consider that the combination of four ultrasonographic criteria improves the diagnostic accuracy to detect malignant thyroid nodules [23–25].

Moon et al. [23] assessed the diagnostic accuracy of US for differentiation between benign and malignant thyroid nodules and found that the US criteria including irregular shape, hypoechogenicity, taller-than-wide dimensions, and microcalcifications, were helpful for the discrimination of benign from malignant thyroid nodules. These criteria had high sensitivity for diagnosing malignant nodule (80%, 81.3%, 75.3%, and 74.7%, respectively). However, this study had a low diagnostic accuracy, which could be explained by the study not using vascularity criteria in their assessment, which proved to be an important feature for the prediction of malignancy in our study.

In the current study, multiple sonographic criteria were assessed for their utility in differentiation between benign and malignant thyroid nodules according to the ATA (American Thyroid Association) guidelines. The criteria that were most predictive of malignancy were the presence of microcalcifications in 11/14 (78.6%) with 93.06% accuracy, internal vascularity or mixture of internal and peripheral vascularity in 12 out of 14 malignant nodules (85.7%) with 88.89% accuracy, absence of a halo around the nodule in 12 out of 14 malignant nodules (85.7%) with 81.94% accuracy, and hypoechogenicity in 10 /14 (71.4%) with 81.94% accuracy. All these criteria are in accordance with the American Thyroid Association (ATA) guidelines and display higher accuracy in the characterization of thyroid nodules in children and adolescents than in adults.

Other criteria were also relatively predictive, albeit with a lower accuracy, including an irregular outline in 8/14 (57.1%) with 80.56% accuracy and longitudinal appearance rather than wideness in 9/14 (64.3%) with 66.67% accuracy. Using a combination of these criteria for the detection of malignancy resulted in 94.2% sensitivity, 69.89% specificity, 74.62% accuracy, 83.21% positive predictive, and 78.9% negative predictive values. These data are in accordance with another study that reported 87.0% sensitivity and 86.5% specificity when using high resolution ultrasonography [10].

Other studies also are concordant with our results [10, 14] and highlight the importance of high-resolution ultrasound (HRUS) in diagnosing thyroid nodules. Rago et al. [26] found that the presence of microcalcifications combined with the absence of a halo sign was highly predictive of malignancy. Takashima et al. [27] concluded that microcalcifications had the greatest accuracy (76%) and highest specificity (93%) for the diagnosis of malignant nodules. However, another report by Shuzhen et al. [28] contradicted these results by showing different results, including a higher sensitivity and a lower specificity of HRUS.

The current study adopted SWE to discriminate between benign and malignant thyroid nodules among children and adolescents as this has not been extensively investigated before. We reported a statistically significant difference among SWE measurements including mean, minimum, and maximum values (*p* < 0.001), highlighting the utility of SWE in this regard, as has been previously documented in adults [13–15].

The cut-off values used as the best index to differentiate benign from malignant thyroid nodules are still under debate, with wide variation between different reports, ranging between a mean valu/e of 85.2 kPa (43.6% sensitivity, 88.7% specificity, 93.8% PPV, and 28.7% NPV [29] and 34.5 kPa (76.9% sensitivity and 71.7% specificity) [15]. Other studies reported SWE cut-off values between 65 kPa with 85.2% sensitivity, 93.9% specificity, 80% positive predictive value, and 95.9% negative predictive value [13] and 66 kPa with 80% sensitivity and 90.5% specificity [14].

The current work found the SWE cut-off value to be 42.2 kPa with the highest accuracy reported yet (93.06%). In accordance with these results, Liu et al. [30] reported an SWE cut-off value of 38.3 kPa for discriminating between benign and malignant nodules.

The discriminative power of SWE proved to be superior to other conventional sonographic tools in detecting malignant thyroid nodule [31]. This notion was clarified in our study as the combination of grayscale and Doppler ultrasound criteria was 80.45% accurate. On the other hand, the accuracy of SWE was 93.06%.

Despite the existence of many sonographic malignancy risk stratification systems, such as ACR-TIRADS, we do recommend considering an SWE of more than 42.2 kPa as a suspicious criterion for malignancy; we also recommend performing FNA for any thyroid nodule classified as TR3 or above according to ACR TI-RADS with an SWE of more than 42.2 kPa.

The diagnostic performance of the combination of the total suspicious sonographic criteria with the mean SWE may be superior to the sole sonographic criteria. However, this combination was variable when compared with the mean SWE (sensitivity 96.3%, specificity 89.8%, accuracy 91.06%, PPV 90.06%, and NPV 88.3%).

Overall, the wide discrepancy between the different reports as regards the SWE cut-off value may be attributed to methodological differences among the studies. ROI application could be the main reason for such differences. Some studies used a fixed size to place it over the stiff part of the nodule to measure the stiffness, while others used different sizes of ROI to examine the stiffness throughout the nodule. Another reason for such variation may be due to the subjective judgment of the operator in placing the ROI. We applied a unified method for the ROI over the stiffest part of the nodule to differentiate the SWE cut-off point both for benign and malignant nodules.

Some limitations existed in the current study, including the low cohort number, which could be explained by low incidence of thyroid nodules in the pediatric age group in our setting. Additionally, the number of malignant nodules was low (14 nodules), and all of them were papillary carcinomas. Hence, we were not able to evaluate the difference of SWE indices among different types of malignant nodules. In addition, not all patients underwent surgical excision, which is the definitive method for diagnosing malignancy, in comparison to FNAB. Despite these limitations, this study contributes by highlighting the efficacy of SWE in the evaluation of children and adolescents’ thyroid nodules.

## Conclusion

SWE is an effective non-invasive technique that can help in the discrimination of benign and malignant thyroid nodules among children and adolescents. A cut-off value of 42.2 kPa may be optimal in predictions of the likelihood of malignancy of thyroid nodules in this age group. Further studies with larger cohorts and standardized sonographic evaluation criteria incorporating SWE may allow the better management of children and adolescent thyroid nodules.

## Data Availability

The datasets used and/or analyzed during the current study are available from the corresponding author on reasonable request.

## Abbreviations

ATA: American Thyroid association
FNAB: Fine needle aspiration biopsy
HRUS: High frequency ultrasound
IRB: Institutional review board
kPa: Kilopascal
SE: Sonoelastography
SWE: Shear wave elastography
